# Co-cultivation effects of *Lactobacillus plantarum* and *Pichia pastoris* on the key aroma components and non-volatile metabolites in fermented jujube juice†

**DOI:** 10.1039/d5ra00193e

**Published:** 2025-04-07

**Authors:** Tao Feng, Weitong Cai, Wei Sun, Shixing Yu, Jianhua Cao, Min Sun, Huatian Wang, Chuang Yu, Wencui Kang, Lingyun Yao

**Affiliations:** a School of Perfume and Aroma Technology, Shanghai Institute of Technology Shanghai 201418 China kangcsong@sit.edu.cn Lyyao@sit.edu.cn; b Hunan Wuzizui Industrial Group Co., Ltd Xiangtan 411228 China

## Abstract

Fermented jujube products are gradually becoming popular. However, few studies have focused on the relationship between the metabolites and aroma compounds in jujube during the fermentation process. Hence, in this study, jujube was fermented with the co-culture of *Lactobacillus plantarum* and *Pichia pastoris*, and the key volatile organic components (VOCs) and non-volatile organic components (nVOCs) in the fermented jujube juice (FJJ) were studied to determine the possible aromatic production pathway during microbial metabolism and propose the possibility of regulating flavor during fermentation. Headspace solid-phase microextraction-gas chromatography-mass spectrometry (HS-SPME-GC-MS) was employed to analyze and compare the VOCs in the jujube juice before and after fermentation, which showed that the fermented aroma had increased floral, winy and sour notes. Specifically, 13 key aroma compounds were found using the aroma extract dilution analysis (AEDA) and aroma recombination/omission model. Additionally, 32 differential nVOC metabolites, mainly involved in amino acid and nucleotide metabolism pathways, were screened in FJJ using liquid chromatography-tandem mass spectrometry (LC-MS/MS) combined with multivariate statistical analysis. After correlation analysis, 14 nVOCs were significantly correlated with 8 key aroma compounds. This study indicates that the combination of *Lactobacillus plantarum* and *Pichia pastoris* may supply a new mixed fermentation agent towards fermented jujube products and provides reference values for flavor regulation in the co-fermentation of jujube juice.

## Abstract 2

Jujube juice was prepared for dynamic fermentation. GC-MS-O, sensory evaluation, and LC-MS identified sensory attribute words and key flavor compounds. Pearson's correlation analysis showed correlations between key aroma compounds and metabolites. The main metabolic pathway of FJJ was plotted.

## Graphical abstract

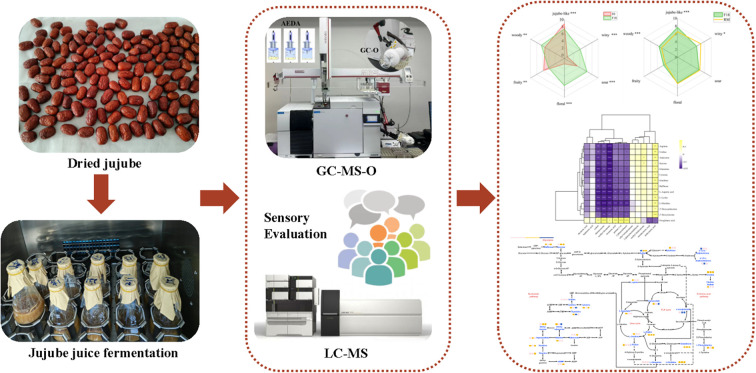

## 1 Introduction

With the great demand for flavor choice and functional attributes of food products, fermented jujube juice (FJJ) has become more and more popular in beverage market and is gaining popularity among consumers.^1^ FJJ can be fermented using mono-culture or mixed strains, resulting in different nutrient and quality features under various inoculum fermentation.^2,3^ Prior to jujube fermentation, some pretreatments, such as enzymatic hydrolysis,^4^ hot water soaking,^5^ and various sterilization methods,^6^ can cause changes in the quality characteristics of FJJ products. During the jujube fermentation process, the microbial strain and environmental factors play important roles in the metabolic profiles and quality features of the FJJ.^7,8^ For example, different lactic acid bacteria (LAB) strains have been found to reveal a clear distinction in both aroma and taste sensations, as reported previously, where *Lactobacillus plantarum* fermented jujube generated more aroma VOCs and exhibited lower bitterness, astringency, and aftertaste.^9^ Additionally, the use of LAB in co-fermentation with acetic acid bacteria (AAB) or yeast has been extensively employed for the preparation of fermented fruit drinks in recent years, which improves the flavor complexity, nutritional value, and shelf-life of the final product.^10,11^ Consequently, the co-fermentation strategy (LAB and AAB) has been successfully adopted for FJJ production owing to its superior capacity in enhancing the functionality and sensory quality of the product.^3,7^

Numerous works have been conducted to screen the potential active components in FJJ in the past few years,^12,13^ whereas the flavor characteristics and key aroma active compounds of the fermented fruit juice have been less investigated.^14^ Flavor is an important factor affecting the sensory perception of food. Under co-cultivation conditions, the synergic interactions between various strains may form higher levels of aroma VOCs in the fermented fruit substrates.^11^ For FJJ, a similar phenomenon was observed during the co-cultivation of *Lactiplantibacillus* and *Streptococcus*. Non-targeted metabolomic analysis and sensory evaluation results revealed that the acids and aroma volatiles were enriched and the characteristics were improved during co-cultivation compared with monoculture fermentation.^14^ Besides LAB and AAB, non-*Saccharomyces* yeasts are well known microbes for the biosynthesis of aroma compounds, which can be employed for fruit juice fermentation.^15^ In recent years, the co-fermentation of LAB with non-*Saccharomyces* yeast has been revealed to be a more efficient method for improving the bioactive capacity and flavor characteristic of fruit-derived non-alcoholic beverages.^16,17^ For example, co-fermentation of *L. plantarum* with *Rhodotorula glutinis* would improve the total phenolic content and antioxidant capacity and increase the norisoprenoid aroma compounds of fermented mango juice.^18^ However, information on the co-cultivation of LAB with non-*Saccharomyces* yeast in terms of metabolite profile and sensory quality of FJJ is still limited.

During the fermentation process, the *Pichia* yeast has considerable advantages among non-*Saccharomyces* yeasts such as lower production of alcohol and accumulation of more aroma substances.^15^ In the present work, the non-*Saccharomyces* yeast *Pichia pastoris* (CGMCC 2.4869) was employed for co-cultivation with two *L. plantarum* strains for FJJ production due to the improved flavor characteristic and acceptability based on preliminary selection. The volatile profile and key aroma compounds of FJJ were characterized by the SPME-GC-MS-O method. Meanwhile, the non-volatile metabolites were identified using LC-MS/MS, and the correlation between metabolites and key aroma compounds was evaluated by Pearson's correlation analysis. The obtained results will provide useful information for understanding and regulating the changes in aroma profile and non-volatile metabolites of FJJ during the co-fermentation process.

## 2 Experiments

### 2.1 Materials and reagents

Dried jujube, from Ruoqiang, Xinjiang, was purchased from the Ecological and Healthy Jujube Garden in Milan Town, Ruoqiang County, Xinjiang. Cellulase (enzyme activity 20 000 U g^−1^) and pectinase (enzyme activity 30 000 U g^−1^) were purchased from Jiangsu Yinong Biotechnology Co., Ltd, China. The detailed information for the other standard chemicals is shown in Table S1.†

### 2.2 Preparation of bacterial strains and FJJ

*L. plantarum* (CICC 21825, *LP*1) was purchased from China Center of Industrial Culture Collection (CICC), while *L. plantarum* (CGMCC 1.12394, *LP*2) and *Pichia pastoris* (CGMCC 2.4869, *PP*) were purchased from China General Microbiological Culture Collection Center (CGMCC), and each strain was cultured according to the product manual. Also, the strains were activated to approximately 10^7^ CFU mL^−1^ for later use.

Jujube was cored with boiling water (1 : 6, g mL^−1^) using a cooking machine and extracted for 30 min to obtain jujube juice (JJ). Then, 0.2% pectinase and cellulase (2 : 1, w/w) were enzymatically hydrolyzed for 120 min at 49.9 °C and the JJ was pasteurized at 90 °C for 20 min. Based on a pre-experiment, *LP*1, *LP*2 and *PP* (1 : 1:1 v/v/v) at a 3% (v/v) inoculation volume were added simultaneously after cooling and fermented under the conditions of stirring at 120 rpm min^−1^ for 18 h at 34 °C.

The total plate count during the fermentation period (0 h, 6 h, 12 h, and 18 h) was determined using the plate counting agar culture method, following the national food safety standard GB 4789.2-2022. The final counting result was in log CFU mL^−1^ (per mL of FJJ). A desktop pH meter was used to directly measure the pH of FJJ during fermentation, taking the average of three measurements each time.

### 2.3 Sensory evaluation

The sensory evaluation test was performed at the Shanghai Institute of Technology. The sensory evaluation method was performed according to a previous study with slight modification.^19^ Before the formal sensory evaluation, the sensory team members received 7 consecutive days of training (1–2 hours per day), mainly to be familiar with the sensory characteristics of JJ before and after fermentation. A consensus was reached on 6 descriptive words to describe the flavor of JJ before and after fermentation, including jujube-like, winy, sour, floral, fruity, and woody. The corresponding compounds were employed to refer to the sensory attributes of these sensory descriptors, namely, methyl dodecanoate, pentan-1-ol, hexanoic acid, β-damascenone, ethyl tetradecanoate, and cedrol, respectively. Each sensory descriptor was scored on an intensity scale of 0–9, with 0 indicating the lowest intensity and no feeling, 9 indicating the strongest intensity and obvious feeling.^20^ This study was reviewed and approved by the School's Institutional Review Board (IRB) and informed consent was obtained from each subject prior to their participation in the study, and the work did not involve experiments on living animals.

### 2.4 Determination of VOCs in FJJ

#### 2.4.1 Extraction of VOCs

The method was performed according to Liu *et al.*,^21^ where 5.00 mL pasteurized FJJ (fermented for 6 h) in a 20 mL headspace bottle and 4 μL 1,2-dicholobenze (100 ppm, soluble in acetone) added as an internal standard. The extraction needle (Supelco®50/30 μmDVB/CAR/PDMS, Sigma-Aldrich, USA) was equilibrated at 60 °C for 10 min, followed by insertion for 30 min in the headspace bottle.

#### 2.4.2 Detection of VOCs based on GC-MS-O

The extraction needle was injected into the GC-MS instrument (GC 8860-MS 5977B, Agilent, Palo Alto, USA) for analysis. An HP-INNOWAX quartz capillary column (60 m × 0.25 mm × 0.25 μm, Agilent Technologies Co., Palo Alto, California, USA) was used for separation, and the column initial temperature was maintained at 40 °C for 2 min, then raised to 85 °C at a rate of 3 °C min^−1^, and finally to 230 °C at 5 °C min^−1^ for 10 min. The carrier gas was helium with a flow rate of 12 mL min^−1^. For the mass spectrometry identification, an EI source with an ionization energy of 70 eV was employed, with the scanning range of 50–550 *m*/*z*. The identification database was NIST 20 (National Institute of Standards and Technology, Gaithersburg, MD, USA).

The GC-O (GC 8860, Agilent, Palo Alto, USA; Olfactory Detection Port ODP4, Gerstel, Mülheim, Germany) programmed temperature conditions were the same as GC-MS, and the aroma description, peak time and aroma intensity of the smelt compounds were recorded during the olfactory detection. The intensity of the aroma was scored on a scale of 0–4, where 0 meant that the aroma was not felt, and 4 meant the aroma was strongly felt.^22^ A sample was sniffed three times.

#### 2.4.3 Aroma extract dilution analysis (AEDA)

To further determine the characteristic VOCs in FJJ, the sample was diluted through AEDA. The method referred to Feng *et al.*^23^ and the dilution multiple modified, that is, the sample volume was diluted by continuously reducing the volume of the sample in the headspace bottle (*i.e.*, 5, 2.5, 1.25 mL…), corresponding to FD value = 2^0^, 2^1^, 2^2^, …, 2^*n*^.

#### 2.4.4 Data analysis of VOCs

All VOCs were evaluated and identified using the NIST 20 spectrum library, the retention index (RI) of the standard compounds, and the olfaction (O) results.^24^

Quantitative analysis was performed using two methods, *i.e.*, semi-quantitative internal standard method and external standard method. The internal standard method referred to Sun *et al.*^25^ The external standard method was described by Zhu & Xiao with modification.^26^ Firstly, dichloromethane was used to extract the fermented juice to gain the tasteless fermented juice matrix. The compound standard with different concentrations (0.023–6.448 mg kg^−1^) was mixed with the internal standard and the volume was fixed with ethanol. Then, the samples were diluted in an odorless matrix at 7 different concentration ratios (1 : 5, 1 : 10, 1 : 50, 1 : 100, 1 : 200, 1 : 400 and 1 : 1000). The peak area of the detected compounds was calculated according to the concentration formula (*A*_*x*_/*A*_*i*_) = *a*(*C*_*x*_/*C*_*i*_) + *b*, where *A* represents the peak area, *C* represents the concentration of each compound, *x* represents the compound standard, *i* represents the internal standard, and *a* and *b* represent the slope of the standard curve of each compound and the intercept on the *Y*-axis, respectively.^24,26^

#### 2.4.5 Threshold determination and OAV calculation of key VOCs

The determination of the thresholds was based on the determination of characteristic VOCs obtained by AEDA in Section 2.4.3, using the forced three-point selection method under the guidance of ISO 13301-2018. The threshold determination of each VOC was repeatedly evaluated three times.^24^ The result was calculated using the correction formula *P* = (3*p* − 1)/2, where *p* is the actual correct identification probability and *P* is the corrected correct identification probability. Furthermore, the threshold of each compound was calculated by establishing the S-curve fitting formula, which was *P* = 1/(1 + exp(−(*x* − *C*)/*D*)), where *x* is the value obtained by logarithmic operation of the configured concentration with base number of 10, *C* is the threshold under the logarithm, and *D* represents the characteristic parameters in the S-curve.^27,28^

The OAV value was used to evaluate the contribution of an aroma compound to the overall aroma of the sample, where generally, the larger the OAV value, the stronger the contribution.^29^ The method for its calculation was the content of a compound obtained after quantification by the external standard method divided by its detection threshold.^24^

#### 2.4.6 Aroma recombination and omission

To verify the key VOCs in the characteristic aroma composition of the FJJ, compounds with calculated OAV ≥ 1 were selected for the aroma recombination and omission test. In brief, these compounds were added to the above-mentioned odorless matrix according to the actual concentration and mixed evenly to obtain the recombination model.^24^ The sensory team members evaluated the recombination model and used a score of 0–9 according to the sensory attributes in Section 2.3.

To assess the critical importance of an aroma compound to its aroma or overall aroma composition, the aroma omission model was used to make a judgment. In simple terms, multiple aroma models were prepared, and each time an entire note or just one aroma compound in one note left out. According to the triangle test (ISO 4120:2021), the combined aroma model and two odorless substrates were formed into one group, and 10 members of the sensory team were selected for testing. The aroma included in the omission/recombination model was similar to the sensory attributes including jujube-like, winy, sour, floral, fruity and woody.

### 2.5 Determination of nVOCs in FJJ

The nVOCs of FJJ were extracted according to the method reported by Chen *et al.*^30^ Firstly, the freeze-dried FJJ was dissolved with 500 μL 70% methanol and centrifuged (12 000 g, 5 min) at 4 °C to obtain the supernatant sample for LC-MS/MS analysis. The analysis for all samples was the same under positive and negative ion conditions. Briefly, in the elution method, 0.1% formic acid was used in water and acetonitrile as solvent A and B, respectively, in the following gradient: 5% A in 2 min, increased to 60% A in the following 3 min, then increased to 99% A in 1 min and held for 1.5 min, and back to 5% B within 0.1 min, and held for 2.4 min through a T3 column (Waters ACQUITY Premier HSS T3 Column 1.8 μm, 2.1 mm × 100 mm). The ion spray voltage floating of MS (Triple TOF 6600+, SCIEX, Foster City, CA, USA) was 5 kV and 4 kV in positive and negative modes, and the source gas ion spray was 50 psi. Also, the data were collected in the ion scan range of 50–1000 *m*/*z*. The original data of non-targeted metabolomics were extracted by XCMS software and corrected for retention time, and then metabolites were identified by the self-built database in the Metware Metabolic platform. Finally, there were 2445 metabolites in positive ion mode and 3830 metabolites in negative ion mode.

### 2.6 Data analysis

The results for all the test samples was expressed in the form of mean ± standard deviation using Office Excel 2011 (Redmond, Washington, USA). ANOVA (analysis of variance) and significance analysis were completed using SPSS 20 (Chicago, USA), while line, bar, radar charts and heatmaps were generated using Origin 2024b (Northampton, Massachusetts, USA).

## 3 Results and discussion

### 3.1 Microbial growth and sensory evaluation of FJJ

**Fig. 1:**
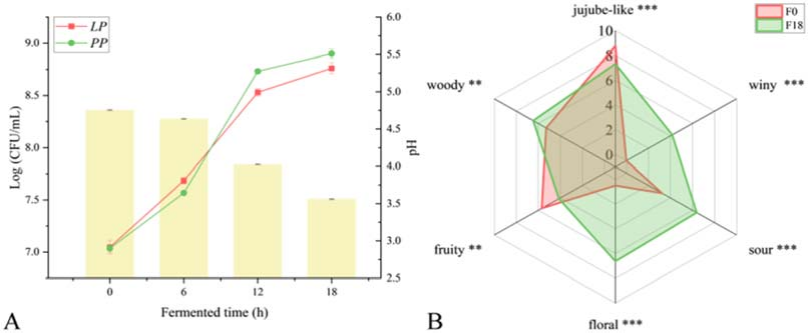
(A) Total plate count of *L. plantarum* and *P. pastoris* (*LP* and *PP*, respectively) and pH values during fermentation. (B) Sensory radar score plot compared with F0 and F18 JJ, “F0”: fermented 0 h, “F18”: fermented 18 h, and “JJ”: jujube juice.

Viable count is a more intuitive method to observe the growth process of microorganisms. As shown Fig. 1A, LAB grew rapidly in the early stage of fermentation (0–6 h), which was similar to the reported observation.^31^ The rich content of fructose and glucose in jujube were high quality carbon sources for the growth and cultivation of LAB, and thus LAB had a higher utilization rate in the early stage of fermentation. In addition, acidification is an essential part of the fermentation process of LAB.^32^ Organic substances in the substrate will be converted by LAB into organic acids (especially lactic acid) and other by-products, thereby reducing the overall pH value of the environment.^33^ Meanwhile, the pH value of the mixed bacteria FJJ showed a slow downward trend in the early stage of fermentation (from 4.75 to 4.65), and then dropped rapidly to about 3.60 in the middle and late stage of fermentation (6–18 h) (Fig. 1A). This may be due to the acidic condition caused by the acid production of LAB, which easily inhibited the growth of LAB. The two kinds of bacteria would compete for the saccharides in JJ,^34,35^ and thus the yeast grew more rapidly in the middle and late stage of fermentation, and the final total colony was close to 9 log (CFU mL^−1^) (Fig. 1A).

Sensory evaluation was conducted on the aroma properties of FJJ for 0 h and 18 h, resulting in a sensory attribute radar chart (Fig. 1B). The results showed that there was a significant change in aroma before and after fermentation. Besides the unique sweet and fruity aroma of jujube itself, which were more pronounced at 0 h of fermentation, all other sensory attributes scored higher after 18 h fermentation (F18) including floral (6.64), sour (6.39), and wine (4.17). Studies have shown that non-*Saccharomyces* have higher glucosidase activity, which can hydrolyze more aroma precursor substances to produce higher alcohols, complex esters, and volatile fatty acid compounds, thereby enriching the aroma characteristics such as floral and winy aroma.^36^

### 3.2 Analysis of key aroma VOCs in FJJ

**Fig. 2:**
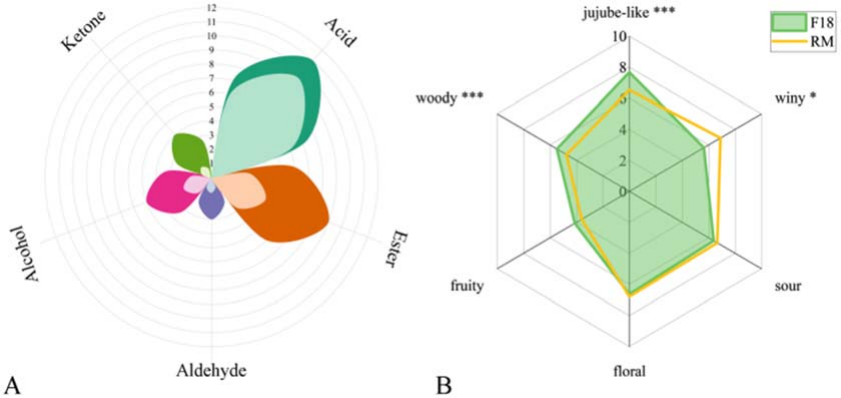
(A) Number of compound types between F0 and F18 JJ and (B) sensory radar score plot compared with F18 jujube juice and recombination model (RM), “JJ”: jujube juice.

A total of 35 compounds was detected in JJ after 0 h and 18 h fermentation, including 13 acids, 9 esters, 4 aldehydes, 5 alcohols and 4 ketones (Table S2†). The number and content of the compound types before and after fermentation showed significant changes (Fig. 2A and Table S2†). It can be clearly seen that JJ itself was rich in a variety of acid and aroma components, given that 9 acid compounds were detected before fermentation (F0), while the content of acids after fermentation (F18) was significantly different from that of F0 (for example, the content of octanoic acid (A7) increased from 0.09 mg mL^−1^ to 0.12 mg mL^−1^, and the content of dodecanoic acid (A11) increased from 0.60 mg mL^−1^ to 6.25 mg mL^−1^). This is because with the fermentation process, the metabolism of LAB continued to produce organic acids and small molecular acids with flavor by consuming sugar-containing substrates.^31^ At the same time, the aroma intensity and AEDA results of F18 are shown in Table 1. Hexanoic acid (A4) and heptanoic acid (A5) had a more pronounced sour aroma and a cheese-like sour odor, while A11 had a strong coconut oil aroma and a soapy feeling.

**Table 1:** VOCs of F18 JJ identified using GC-MS-O and AEDA^a^

No.	Compound	CAS no.	RI	Odor description	Aroma intensity	FD value	Identification method
A	Acid						
A4	Hexanoic acid	142-62-1	1831	Sour cheese	2	8	MS, RI, O, Std
A5	Heptanoic acid	111-14-8	1950	Rancid sour	2	4	MS, RI, O, Std
A8	Decanoic acid	334-48-5	2265	Sour rancid	2	2	MS, RI, O, Std
A11	Dodecanoic acid	143-07-7	2503	Oily coconut soapy	2	8	MS, RI, O, Std
A13	Tetradecanoic acid	544-63-8	2713	Waxy fatty	1	4	MS, RI, O, Std

B	Ester						
B1	Methyl decanoate	110-42-9	1604	Sweet fruity winy	2	2	MS, RI, O, Std
B3	Methyl dodecanoate	111-82-0	1815	Waxy fatty oily	1	8	MS, RI, O, Std
B5	Methyl tetradecanoate	124-10-7	2020	Warm waxy	1	1	MS, RI, O, Std
B7	Ethyl tetradecanoate	124-06-1	2057	Sweet waxy floral	3	8	MS, RI, O, Std
B8	Methyl hexadecanoate	112-39-0	2226	Oily sweet floral	2	1	MS, RI, O, Std

C	Alcohol						
C1	Pentan-1-ol	71-41-0	1244	Fermented alcohol	2	2	MS, RI, O, Std
C4	2-Phenylethanol	60-12-8	1872	Like sweet rose	3	32	MS, RI, O, Std
C5	(1*R*,2*R*,5*R*,7*S*,8*R*)-2,6,6,8-Tetramethyltricyclo[5.3.1.01,5]undecan-8-ol	77-53-2	2149	Woody sweet	1	4	MS, RI, O, Std

D	Aldehyde						
D3	2-Phenylacetaldehyde	122-78-1	1663	Strong green floral	4	16	MS, RI, O, Std

E	Ketone						
E2	(*E*)-1-(2,6,6-Trimethyl-1,3-cyclohexadien-1-yl)-2-buten-1-one	23726-93-4	1831	Rose honey floral	3	64	MS, RI, O, Std
E3	Pentadecan-2-one	2345-28-0	2025	Fresh green floral	1	1	MS, RI, O, Std
E4	6,10,14-Trimethylpentadecan-2-one	502-69-2	2131	Oily	1	1	MS, RI, O, Std

a Odor description was detected at the sniffing port of ODP4; FD value was flavor dilution value; MS, compounds were identified using MS spectra: NIST 20; RI, compounds were identified through calculated value and searched value; O, compounds were identified by comparison of their odor with that of authentic compounds using GC-O; and S, compounds were identified by comparison with standards.

The formation of ester compounds was mainly due to the non-enzymatic esterification reaction of alcohols produced by yeast metabolism and organic acids produced by LAB,^37^ which were considered to be the main class of compounds with a fruity aroma. F0 contained methyl dodecanoate (B3), methyl tetradecanoate (B5) and methyl hexadecanoate (B8). Based on fruity, they also have the special fat wax fragrance and oil fragrance of jujube, which are the representative compounds in Ruoqiang jujube,^38^ and their content increased in F18. The olfaction results in Table 1 show that the FD value of B3 is higher, which is described as having the aroma of fatty, waxy and sweet. In addition, methyl decanoate (B1) and ethyl tetradecanoate (B7) were newly produced after fermentation, and although their content was low, they could still be detected many times in the olfactory results (FD = 2 and 8). B1 was fruity and winy, while B7 had a more floral note.

Alcohols are mainly metabolites of yeast bacteria. They are produced by amino acids through decarboxylation and dehydrogenation pathways.^39^ For example, phenylmethanol (C3) and 2-phenylethanol (C4) are produced by the phenylalanine pathway. However, C4 (FD = 32) could be clearly perceived in the olfactory results, which had a rose sweet aroma, while C3 could not be perceived due to its low content. In addition, pentan-1-ol (C1) and its isomers are important fermentation flavor compounds in the food industry. They are often found in the flavor compounds of liquor, which endow it with a full-bodied aroma and mainly generated through the biosynthetic pathway of leucine or valine.^40^ C1 was considered to have ethanol-like translucency and fermented aroma in smell, but due to its certain volatility, its FD value was only 2.

Aldehyde compounds are easily oxidized or reduced, mainly through the oxidation of amino acids or fatty acids. Nonanal (D1), 2-phenylacetaldehyde (D3) and 2-benzylideneoctanal (D4) were newly generated aldehydes with floral fragrance in F18. D1 had a thick sense of rose fragrance and lipid-like fragrance, and D3 had a floral fragrance like hyacinth.^41^ However, only the aroma characteristics of D3 (FD = 16) were felt, as shown in Table 1. Ketones and aldehydes are formed in a similar way and obtained by the degradation of their precursor amino acids or the transformation of alcohols. 6,10,14-Trimethylpentadecan-2-one (E4) was the original of F0 and its content increased slightly with the fermentation process. It had an oily fragrance and a slightly sweet floral fragrance,^39^ where (*E*)-1-(2,6,6-trimethyl-1,3-cyclohexadien-1-yl)but-2-en-1-one (known as β-damascenone) (E2) was the ketone compound with the highest content in F18 (0.3367 mg mL^−1^), which is produced by yeast metabolism and considered to be an important aroma component of wine beverages. It had a strong rose-like sweet and honey-like sweet fragrance.^39^ Because of its low threshold value and high aroma vitality value, it had a high value of aroma activity, and also its FD factor was as high as 64 in the diluted smell, which was the compound with the highest FD value in F18, and this it was initially considered to be an important aroma compound in sweet floral fragrance. Moreover, 17 VOCs with FD factors were subjected to the use of an external standard and threshold detection analysis, as shown in Table S3.†

To further determine the key aroma compounds in mixed bacteria FJJ, the aroma characteristics of F18 were simulated by aroma recombination and omission experiments. Compounds with FD ≥ 2 and OAV ≥ 1 were selected and mixed in the odorless matrix according to the actual concentration to obtain the recombination model of F18. The sensory group scored and evaluated the attributes of each group, and the results are shown in Fig. 2B. Compared with F18, the recombination model had significantly lower scores for jujube and woody, slightly lower scores for fruity, and higher scores for winy. Only the scores of floral and sour notes were similar to F18. Jujube-like and woody note were considered to be close to the original dried jujube and the incense similar to jujube kernel when the sensory attributes were first proposed. These aroma features were derived from nature and cannot be obtained simply by mixing several monomer aroma compounds. This may require synergistic effects between a variety of aroma compounds. Overall, the aroma recombination model showed good similarity compared to F18.

The compounds in the omission model were classified by aroma note, and the aroma omission test was carried out by omitting a group of notes or a single compound in one note, and the results are shown in Table 2. Similar to the aroma recombination model, the group members were more sensitive to floral and sour notes, especially hexanoic acid (3-1), octanoic acid (3-2), β-damascenone (4-1) and 2-phenylethanol (4-2). In the jujube-like note, the absence of dodecanoic acid (1-1) and tetradecanoic acid (1-3) was perceived by most members. The description of aroma loss mainly mentioned the absence of fatty or lipid waxy note. Some studies concluded that the main characteristic aroma of jujube is fatty and fruity, and its aroma is not only provided acid compounds with more than 10 carbon atoms, but also conferred by aldehydes such as nonan-2-al and decan-2-al.^42,43^ However, although the OAV values of phenylacetaldehyde (4-3) and cedrol (6) were >10, they were not obviously felt in the omission model, which was related to the lower threshold of 4-1 and 4-2 and their high OAV values (OAV = 39 828 and 3302) in the floral fragrance omission model, respectively, while the loss of woody note was not easily felt by members. This may be due to the fact that the content of cedrol (6), represented by woody aroma, was relatively low in the overall composition, which was easily masked by the winy compound pentan-1-ol (2-1). At the same time, methyl decanoate (4-1) in the fruit aroma also had the aroma of fruity and winy, which was also the reason why the winy aroma score, as shown in Fig. 2B, was higher than the actual value. In conclusion, as further confirmed by the aroma recombination/omission model, dodecanoic acid, tetradecanoic acid, methyl dodecanoate, pentan-1-ol, hexanoic acid, heptanoic acid, β-damascenone, 2-phenylethanol, methyl decanoate, and cedrol were the most important aroma compounds in FJJ, and the compounds with FD ≥ 2 and OAV ≥ 1 were phenylacetaldehyde, decanoic acid and methyl tetradecanoate, which also contributed to the overall aroma.

**Table 2:** Result of aroma omission model of F18 JJ^a^

Constructed flavor note	Model no.	Omitted odorants	Difference in odor	Number of correct answers
Jujube-like note	1	Jujube-like waxy	Less waxy and soapy	6*
	1-1	Dodecanoic acid	Lack of soap and crayon fragrance	9**
	1-2	Methyl dodecanoate	Less fatty	5*
	1-3	Tetradecanoic acid	Lack of oily smell	8**
Fermented note	2	Pentan-1-ol	Lacking the aroma of wine and ethanol	5*
Sour note	3	Sour	Lacking the sour aroma of nasal stimulation	9**
	3-1	Hexanoic acid	The lack of sharp thorns in the acid, the grassy aroma is heavy	7**
	3-2	Heptanoic acid	Sharp acid deficiency is more pronounced	9**
	3-3	Decanoic acid	Slightly weak sour aroma	2
Floral note	4	Floral	Without floral and sweet	7**
	4-1	(*E*)-1-(2,6,6-Trimethyl-1,3-cyclohexadien-1-yl)-2-buten-1-one (β-damascenone)	Without the sweet fragrance of flowers	9**
	4-2	2-Phenylethanol	Less floral fragrance with a hint of green leaves	6*
	4-3	2-Phenylacetaldehyde	The sweet fragrance of flowers still lingers, and one cannot feel the lack	2
Fruity note	5	Fruity	Less sweet but different with floral	4*
	5-1	Methyl decanoate	Less winy	4*
	5-2	Ethyl tetradecanoate	Less some sweet	1
Woody note	6	(1*R*,2*R*,5*R*,7*S*,8*R*)-2,6,6,8-Tetramethyltricyclo[5.3.1.01,5]undecan-8-ol (cedrol)	Less smoked woody	3

a **Highly significant (*p* ≤ 0.01); *significant (*p* ≤ 0.05); and not significant (*p* > 0.05).

### 3.3 Differential analysis of characteristic nVOCs in FJJ

**Fig. 3:**
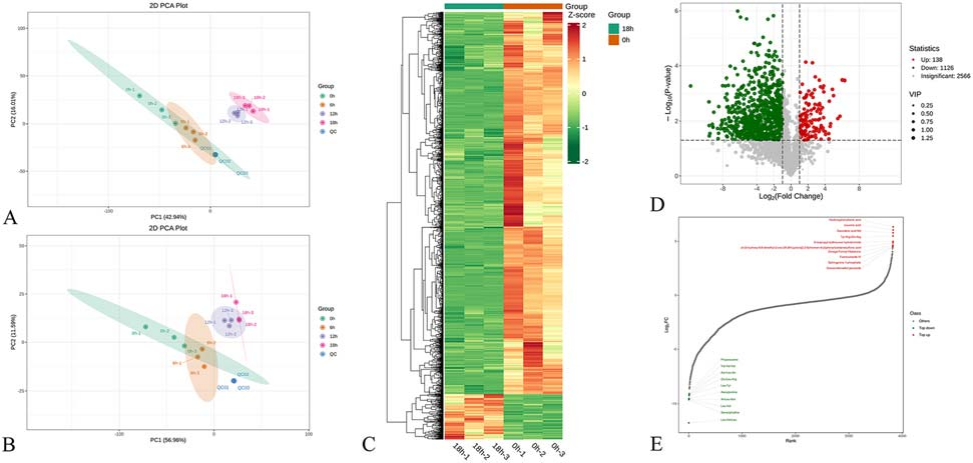
(A) PCA plot in pos ion mode; (B) PCA plot in neg ion mode; (C) heatmap of nVOCs of F0 and F18 of FJJ; (D) volcano plot of nVOCs compared F0 with F18; and (E) nVOCs with top 20 FC values between F0 and F18 of FJJ.

To study the difference and key metabolites during the fermentation process, the fermented juice was sampled every six hours, centrifuged and frozen. The grouped samples were detected by LC-MS/MS, and the data were sorted by multivariate statistical analysis to finally obtain the key differential metabolites before and after fermentation. Before the difference analysis, principal component analysis (PCA) was performed on the grouped samples to observe the overall variation between and within groups of the grouped samples.^44^ As shown in Fig. 3A and B, the PCA analysis results of the two modes are ideal. The QC sample points were used to monitor the stability of the instrument, and the three parallel and close overlaps indicate that the instrument had good stability and repeatability.^44^ The principal component contribution rate of the positive ion mode (Fig. 3A) was close to 60%. In the negative ion mode (Fig. 3B), F0 and F6 overlapped, indicating that the metabolite content slightly changed. Similarly, F12 and F18 were close to each other, indicating that the juice fermented for four different periods was clustered into two groups.

To further separate the samples obtained at different periods, the supervised multivariate statistical analysis method OPLS-DA was selected to distinguish samples from different groups, and 200 permutation tests were used to verify the feasibility of the fitting model and the VIP value was calculated to identify the key variables affecting the classification of groups.^45^ As shown in Fig. S1,† the four groups of samples were grouped in pairs and had good separation. In the permutation test (Fig. S2†), the *Q*^2^ and *R*^2^*Y* values were close to 1, and no over-fitting phenomenon was shown, indicating that the OPLS-DA model was effective and had good predictive ability, which could be used to predict the differential metabolites of FJJ before and after fermentation. Therefore, in the subsequent difference analysis, we mainly focused on the difference between F0 and F18.

The variable importance in the projection (VIP) value reflects the importance of a variable in the projection model, and its size is used to measure the contribution of each metabolite to the classification ability of the model, which is an important parameter in OPLS-DA. Usually, a VIP value of >1 is considered a differential metabolite. In differential analysis, VIP > 1 and *p* < 0.05 are commonly used to determine the differential metabolites between groups.^44,46^ The metabolic substances with VIP > 1 and *p* < 0.05 were screened in the OPLS-DA model before and after fermentation (F0 *vs.* F18), and 1264 substances were detected, among which 138 were up-regulated and 1126 were down-regulated. The hierarchical clustering heat map and volcano map (Fig. 3C and D, respectively) were used to more intuitively observe the relative content changes in the metabolites before and after fermentation. As shown in Fig. 3C, darker red represents a greater content of this substance, while darker green represents less of this substance, and as shown in Fig. 3D, red represents the up-regulated substance, and green represents the down-regulated substance. Both showed that most of the metabolites were consumed after fermentation, with only nearly one-tenth of the metabolites showing an increase. In addition, the fold change (FC) refers to the ratio of the expression level of metabolites in F0 and F18, which can reflect the change fold in the expression level of a metabolite before and after fermentation. The bar chart in Fig. 3E shows the top 20 metabolites with the largest log_2_ FC absolute values among the 1264 metabolites. Most of the down-regulated metabolites were amino acids and their derivatives. Jujube is rich in a variety of free amino acids, including eight amino acids necessary in the human body. Men *et al.* studied the changes in the nutritional composition of JJ before and after fermentation by enzymatic hydrolysis and *L. plantarum* mixed with *Pediococcus pentose*.^47^ The results showed that this fermentation method could increase the production of GABA and increase the content of branched chain amino acids and free amino acids, such as aspartate, glutamic acid, and proline, which provides a theoretical basis for LAB fermentation of JJ to improve the content of nutrients. Liu *et al.* pointed out that amino acids were the energy source for the growth of LAB, and the content of free amino acids in FJJ decreased significantly.^48^ In addition, in the study on the single yeast fermentation of fruit wine, it was found that the concentration of amino acids or ammonium ions would affect the growth and metabolism rate of yeast, and eventually reduce the possibility of ethanol metabolism and affect the flavor and taste of fruit wine.^49^

### 3.4 KEGG pathway enrichment analysis of the differential metabolites

**Fig. 4:**
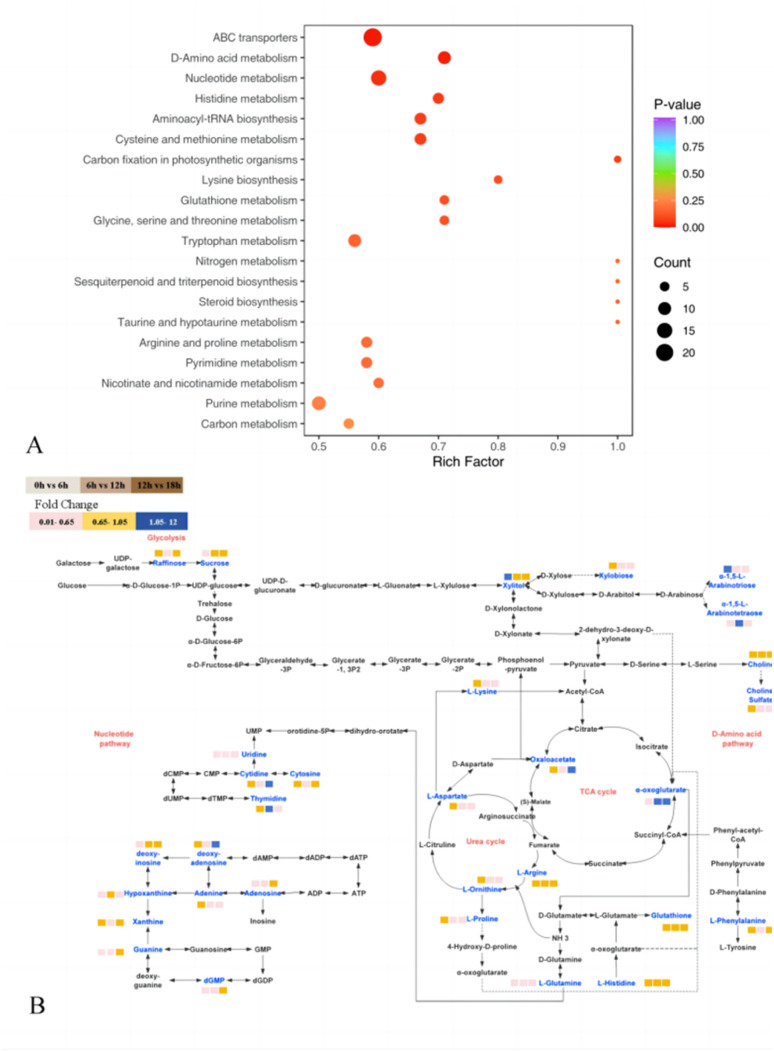
(A) KEGG enrichment analysis of differential metabolite pathways in 0 h *vs.* 18 h and (B) metabolic pathways of major nVOCs during the fermentation of JJ from 0 h to 18 h. “JJ”: jujube juice.

In the KEGG enrichment pathway analysis (Fig. 4A), the main pathways with significant differences (*p* < 0.05) were ABC transporter, amino acid metabolism and nucleotide metabolism, among which the metabolic pathways were similar to some previous studies.^42,47,49^ ABC transporters are an important class of membrane transporters, which mainly drive the transport of various substances across the membrane by hydrolyzing ATP and considered enzyme or transporter.^50^ Through the search of the KEGG enrichment pathways, we sorted and plotted the main metabolic pathways of 32 metabolites together (Fig. 4B), and combined the KEGG metabolic pathways with OPLS-DA and analyzed the findings together (Table S4†). Finally, 14 metabolites out of 32 involved in the major metabolic pathways were identified as key differential metabolites (VIP ≥ 1, *p* ≤ 0.05).

### 3.5 Correlation analysis between key metabolites and key aroma components

**Fig. 5:**
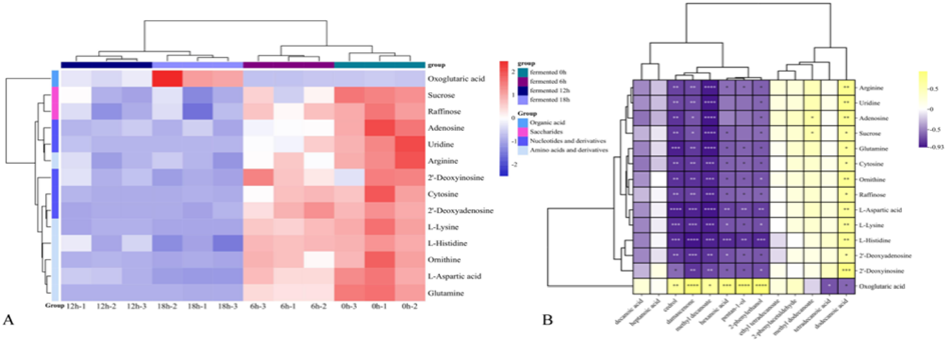
(A) Relative content heatmap of 14 selected key nVOCs of FJJ and (B) heatmap representation of Pearson's correlation analysis of selected differential nVOCs and key VOCs.

As shown in the cluster heat map in Fig. 5A, 14 differential metabolites had obvious content changes in different fermentation periods. In the early fermentation period (F0 and F6), these metabolites were rapidly used and metabolized by LAB and yeast, while in the late fermentation period (F12 and F18), due to the decline in the growth rate of LAB, most of the metabolites had a similar content distribution. Only α-ketoglutarate acid continued to accumulate during the later stages of fermentation. Previous studies have shown that α-oxaloacetic acid (α-ketoglutarate acid) is produced through the glycolysis or phosphoketolase pathway to pyruvate, and then enters the tricarboxylic acid cycle to be transformed by citrate.^51,52^ At the same time, in the tricarboxylic acid cycle, many amino acids are metabolites of citrate and α-ketoglutarate acid under the action of transaminases. For example, valine and leucine with bitter taste were converted to α-ketoglutarate acid during the fermentation process of LAB, and then more aldehydes, acids and alcohols were generated under the action of some dehydrogenases, thus enriching the aroma after fermentation.^53^ Aspartic acid, an amino acid with a sour and umami taste, may change the metabolism of d-glucose and lead to the excessive production of acetic acid, which in turn can combine with alcohols produced by yeast to produce esters with fruity taste.^54^ Therefore, the tricarboxylic acid cycle is an important metabolic process in microbial fermentation. In addition, the key nucleotides involved in the KEGG metabolic pathway are mainly uridine, adenosine and deoxyinosine. The reason for the reduction in these nucleotides may be the salvage pathway occurring in the fermentation process. Due to the continuous decomposition of nutrients in the substrate, a series of specific enzymes could be used to recycle nucleoside substances, with the purpose of reducing the high energy consumption of microorganisms. Simply, under certain conditions, nucleoside compounds can be used as alternative energy sources to support cell growth in an environment where glucose is completely lacking, and microbial metabolic pathways support ATP energy production.

To better illustrate the influence of fermentation of LAB and yeast on the aroma profile of FJJ, a correlation analysis between key selected metabolites and key aroma components was also performed. As shown in Fig. 5B, a total of 8 key aroma components was significantly correlated with 14 key selected metabolites. It is not difficult to find that α-ketoglutarate acid was positively correlated with most aroma components. α-Ketoglutarate acid is produced by glutamate dehydrogenase acting on glutamate, and it is also a key node connecting carbon-nitrogen metabolism in cells (Fig. 5B).^52^ As an intermediate metabolite, α-ketoglutarate acid can indirectly participate in the formation of volatile aroma substances. It can also be found in Fig. 5B that nucleotide and amino acid compounds were negatively correlated with most of the aroma components, which also verified that the metabolic decomposition of amino acids and nucleotides during fermentation was the main metabolic pathway and may indirectly produce VOCs with aroma.

As shown in Fig. 5B, histidine, leucine, and aspartic acid showed relatively significant negative correlations with most aroma components, while decanoic acid, heptanoic acid, ethyl tetradecanoate, and phenylacetaldehyde may not be significantly correlated due to their low content. Previous studies have shown that LAB such as *L. plantarum* and *L. acidophilus*, which contain more genomic fragments, can participate in different metabolic pathways to produce flavor compounds through the action of enzymes and other functional proteins encoded by the genome on amino acids.^7,53^ Research on the metabolic pathways of yeast used in alcohol fermentation shows that amino acids are metabolized in yeast by the Ehrlich pathway and produce higher alcohols and other aromatic substances.^55^ In the process of nucleotide metabolism, LAB and yeast could obtain the corresponding nucleoside through the dephosphorylation of nucleotidases contained in microorganisms.^56^ As shown in Fig. 5B, the main nucleosides negatively correlated with methyl decanoate, cedrol, β-damascenone, hexanoic acid and pentan-1-ol were uridine, cytidine, inosine and cytosine, respectively. Overall, these metabolites are complex and the provided conditions produced volatile aroma components during the fermentation of JJ.

## 4 Conclusions

In summary, the mixed fermentation of *L. plantarum* and *P. pastoris* could not only increase the total plate count of FJJ, but also enrich its aroma and flavor. Through GC-MS external standard quantification and sensory analysis, the key aroma components of FJJ were determined to be dodecanoic acid, tetradecanoic acid, methyl dodecanoate, pentan-1-ol, hexanoic acid, heptanoic acid, β-damascenone, 2-phenylethanol, methyl decanoate, cedrol, phenylacetaldehyde, decanoic acid and methyl tetradecanoate. These aroma components endowed FJJ with jujube-like, winy, sour, floral, woody, and fruity aromas. In addition, through untargeted metabolomics analysis, it was found that the main metabolic pathways of co-fermentation of JJ were amino acid and nucleotide metabolism. Ultimately, 14 key non-volatile metabolites were identified in FJJ at four fermentation periods, among which, cedrol, β-damascenone, methyl decanoate, pentan-1-ol, 2-phenylethanol, and dodecanoic acid were significantly correlated with the key metabolites. This study demonstrates the possibility of regulating fermentation flavor, but the metabolite pathways are complex, and the enzymes involved in the reaction are diverse. Thus, further bioengineering research is needed to confirm the relationship between aroma components and metabolites.

## Data availability

All data included in this study are available from the corresponding author upon request.

## Author contributions

Tao Feng: conceptualization, funding acquisition, project administration, supervision, writing-review & editing. Weitong Cai: data curation, formal analysis, methodology, software, writing-original draft. Wei Sun: conceptualization, project administration. Shixing Yu: conceptualization, project administration. Jianhua Cao: conceptualization, project administration. Min Sun: supervision, validation. Huatian Wang: supervision, validation. Chuang Yu: methodology, visualization. Wencui Kang: visualization, writing-review & editing. Lingyun Yao: conceptualization, visualization, writing-review & editing.

## Conflicts of interest

The authors declare that they have no known competing financial interests or personal relationships with other people or organizations that could inappropriately influence the work reported in this paper.

## Supplementary Material

RA-015-D5RA00193E-s001

## References

[cit1] Rashwan K., Karim N., Shishir M. R. L., Bao T., Lu Y., Chen W. (2020). J. Funct. Foods.

[cit2] Cai W., Tang F., Shan C., Hou Q., Zhang Z., Dong Y., Guo Z. (2020). Food Sci. Nutr..

[cit3] Boasiako T. A., Yinka A. A., Yuqing X., Boateng I. D., Ma Y. (2024). Food Biosci..

[cit4] Zhang L., Zha M., Li S., Zong W. (2022). J. Food Meas. Char..

[cit5] Liu G., Wu M., Li Y., Qayyum N., Li X., Zhang J., Wang C. (2023). LWT--Food Sci. Technol..

[cit6] Zhang L., Zha M., Li S., Zong W. (2022). J. Food Sci. Technol..

[cit7] Pan X., Zhang S., Xu X., Lao F., Wu J. (2022). LWT--Food Sci. Technol..

[cit8] Li H., Fan L., Yang S., Lei W., Tan P., Liang J., Gao Z. (2024). Food Biosci..

[cit9] Cai W., Tang F., Zhao X., Guo Z., Zhang Z., Dong Y., Shan C. (2019). J. Food Process. Preserv..

[cit10] Toy J. Y. H., Lu Y., Huang D., Matsumura K., Liu S. (2022). Crit. Rev. Food Sci. Nutr..

[cit11] Rajendran S., Silcock P., Bremer P. (2023). Molecules.

[cit12] Li T., Jiang T., Liu N., Wu C., Xu H., Lei H. (2021). Food Chem..

[cit13] Ren W., Ma Y., Liu D., Liang P., Du J., Yang S., Tang L., Wu Y. (2022). J. Food Sci..

[cit14] Li J., Xu H., Li H., Xie Y., Ding K., Xu S., Wang Z., Wang R., Yi C., Ding S. (2024). Food Res. Int..

[cit15] Bao Y., Zhang M., Chen W., Chen H., Chen W., Zhong Q. (2021). Food Biosci..

[cit16] Li Y., Nguyen T. T. H., Jin J., Lim J., Lee J., Piao M., Mok I., Kim D. (2021). Food Sci. Biotechnol..

[cit17] Nyhan L., Sahin A. W., Arendt E. K. (2023). Eur. Food Res. Technol..

[cit18] Zhong Q., Chen R., Zhang M., Chen W., Chen H., Chen W. (2023). Fermentation.

[cit19] Wang Z., Xiao Q., Zhuang J., Feng T., Ho C. T., Song S. (2019). J. Agric. Food Chem..

[cit20] Chen Y., Huang Y., Bai Y., Fu C., Zhou M., Gao B., Wang C., Li D., Hu Y., Xu N. (2017). LWT--Food Sci. Technol..

[cit21] Liu Y., Sang Y., Guo J., Zhang W., Zhang T., Wang H., Cheng S., Chen G. (2021). Food Sci. Nutr..

[cit22] Wang L., Wang P., Deng W., Cai J., Chen J. (2019). LWT--Food Sci. Technol..

[cit23] Feng Y., Cai Y., Sun-Waterhouse D., Cui C., Su G., Lin L., Zhao M. (2015). Food Chem..

[cit24] Song W., Sun M., Lu H., Wang S., Wang R., Shang X., Feng T. (2024). Foods.

[cit25] Sun Y., Zhang Y., Song H. (2020). J. Food Process. Preserv..

[cit26] Zhu J., Xiao Z. (2018). J. Agric. Food Chem..

[cit27] Lytra G., Tempere S., Le Floch A., de Revel G., Barbe J. C. (2013). J. Agric. Food Chem..

[cit28] Yang Y., Yu P., Sun J., Jia Y., Wan C., Zhou Q., Huang F. (2022). Food Chem..

[cit29] Du X., Plotto A., Baldwin E., Rouseff R. (2011). J. Agric. Food Chem..

[cit30] Chen S., Liu H., Zhao X., Li X., Shan W., Wang X., Yu X. (2020). Food Res. Int..

[cit31] Zhao M., Zhang F., Zhang L., Liu B., Meng X. (2019). Int. J. Food Sci. Technol..

[cit32] Gao Q., Wu C., Wang M. (2013). J. Agric. Food Chem..

[cit33] Fu W., Mathews A. (1999). Biochem. Eng. J..

[cit34] Gobbetti M., Corsetti A., Rossi J. (1994). Appl. Microbiol. Biotechnol..

[cit35] Liao Y., Ao X., Kang H., He T., Yang L. (2023). Food Ferment. Ind..

[cit36] Wang X., Fan G., Peng Y., Xu N., Xie Y., Zhou H., Liang H., Zhan J., Huang W., You Y. (2023). J. Food Compos. Anal..

[cit37] Jiang X., Peng D., Zhang W., Duan M., Ruan Z., Huang S., Zhou S., Fang Q. (2021). Food Chem..

[cit38] Gou M., Chen Q., Qiao Y., Jin X., Zhang J., Yang H., Fauconnier M. L., Bi J. (2023). J. Food Compos. Anal..

[cit39] Chen Q., Song J., Bi J., Meng X., Wu X. (2018). Food Res. Int..

[cit40] Wang L., Zhu L., Zheng F., Zhang F., Shen C., Gao X., Sun B., Huang M., Li H., Chen F. (2021). J. Food Sci..

[cit41] Fu W., Ren J., Li S., Ren D., Li X., Ren C., Zhao X., Li J., Li F. (2023). Foods.

[cit42] Jia Y., Wang C., Zhang Y., Deng W., Ma Y., Ma J., Han G. (2024). Foods.

[cit43] Liu R., Ma L., Meng X., Zhang S., Cao M., Kong D., Chen X., Li Z., Pang X., Bo W. (2024). Plants.

[cit44] Ai J., Wu Q., Battino M., Bai W., Tian L. (2021). Food Chem..

[cit45] Huang B., Zha Q., Chen T., Xiao S., Xie Y., Luo P., Wang Y., Liu L., Zhou H. (2018). Phytomedicine.

[cit46] Kang C., Zhang Y., Zhang M., Qi J., Zhao W., Gu J., Guo W., Li Y. (2022). Food Chem..

[cit47] Men Y., Zhu P., Zhu Y., Zeng Y., Yang J., Sun Y. (2019). Food Sci. Nutr..

[cit48] Liu W., Luo X., Qiu S., Huang W., Su Y., Li L. (2023). BMC Microbiol..

[cit49] Mas A., Guillamon J. M., Torija M. J., Beltran G., Cerezo A. B., Troncoso A. M., Garcia-Parrilla M. C. (2014). BioMed Res. Int..

[cit50] Higgins C. F. (1992). Annu. Rev. Cell Biol..

[cit51] De Vuyst L., Leroy F. (2019). FEMS Microbiol. Rev..

[cit52] Xiao T., Khan A., Shen Y., Chen L., Rabinowitz J. D. (2022). Nat. Chem. Biol..

[cit53] Ardö Y. (2006). Biotechnol. Adv..

[cit54] Vasserot Y., Dion C., Bonnet E., Maujean A., Jeandet P. (2001). J. Appl. Microbiol..

[cit55] Kim B., Cho B. R., Hahn J. S. (2014). Biotechnol. Bioeng..

[cit56] Kilstrup M., Hammer K., Ruhdal Jensen P., Martinussen J. (2005). FEMS Microbiol. Rev..

