# Supplementary material for: Co-cultivation effects of Lactobacillus plantarum and Pichia pastoris on the key aroma components and non-volatile metabolites in fermented jujube juice
Source: RSC Adv. 2025 Apr 7;15(14):10653–62. doi: 10.1039/d5ra00193e (PMC11973478; doi:10.1039/d5ra00193e)
Supplement: RA-015-D5RA00193E-s001 [file RA-015-D5RA00193E-s001.pdf]

Supplementary Information (SI) for RSC Advances.
This journal is © The Royal Society of Chemistry 2025

Supplementary materials

Table S1 Source information of chemical standards

Chemical Standards	Source
N-alkanes (C7 ~ C30) standard solution ,dissolved in n-hexane	Merck Supelco® Co., New Jersy, USA
1,2-dicholobenze (>99.8%), 6,10,14-trimethylpentadecan-2-one (≥ 98%)	Boer, Shanghai, China
sodium chloride	Hushi, Shanghai, China
pentan-1-ol (99%), hexanoic acid (99%), heptanoic acid (99%), decanoic acid (99%), 2-phenylacetaldehyde (≥ 95%), 2-phenylethanol (≥ 98%), ethyl tetradecanoate (99%), methyl dodecanoate (≥ 98%), methyl 14-methylpentadecanoate (99%), dodecanoic acid (99%), cedrol (99%)	Adamas Beta, Shanghai, China
methyl decanoate (99%), methyl tetradecanoate (≥ 95%), pentadecan-2-one (≥ 95%), tetradecanoic acid (99%)	Meryer, Shanghai, China
β-damascenone (≥ 98%)	Dr. Ehrenstorfer, Augsburg, Germany

Table S2 VOCs identified by GC-MS in F0 and F18 jujube juice

NO.	Compound	RI (HP-INNOWAX)	RI (caculated)	Odor	Identification method	F18 Concentraion (mg/mL)	F0 Concentraion (mg/mL)	p value
A	Acid							
A1	propanoic acid	1526	1565.01	cheesy vinegar	MS, RI	0.0258±0.0023	-	0.000039
A2	butanoic acid	1639	1631.56	acetic cheese	MS, RI	-	0.0038±0.0004	0.004608
A3	pentanoic acid	1734	1739.31	acidic sweaty rancid	MS, RI	-	0.0069±0.0015	0.014935
A4	hexanoic acid	1831	1867.72	sweet waxy floral soapy	MS, RI	0.1252±0.0108	0.1032±0.0067	0.039878
A5	heptanoic acid	1950	1975.19	sour fatty sweat	MS, RI	0.1263±0.0188	0.0793±0.0024	0.01261
A6	(Z)-tetradec-9-enoic acid	2021	2032.05	rancid sour cheesy	MS, RI	0.6763±0.0865	-	0.005406
A7	octanoic acid	2039	2082.62	waxy	MS, RI	0.1187±0.0109	0.0958±0.0024	0.023546
A8	decanoic acid	2265	2294.74	rancid oily vegetable	MS, RI	2.3137±0.2013	0.3874±0.0228	0.00008
A9	undecanoic acid	2401	2400.48	unpleasant rancid sour	MS, RI	0.3488±0.0132	0.0046±0.0017	0.000403

A10	benzoic acid	2448	2482.68	waxy creamy cheese	MS, RI	0.6097±0.1077	-	0.010236
A11	dodecanoic acid	2503	2505.83	faint balsam urine	MS, RI	6.2478±0.3282	0.5968±0.0616	0.000008
A12	3-phenylpropanoic acid	2650	2666.30	mild fatty coconut	MS, RI	0.3023±0.0817	-	0.023496
A13	tetradecanoic acid	2713	2740.27	sweet fatty cinnamon	MS, RI	1.0299±0.1318	0.0558±0.0015	0.000215
B	Ester							
B1	methyl decanoate	1604	1602.19	oily wine fruity	MS, RI	0.0248±0.0009	-	0.000001
B2	benzyl acetate	1742	1746.67	sweet floral fruity	MS, RI	0.0105±0.0015	-	0.006955
B3	methyl dodecanoate	1815	1809.63	waxy soapy creamy	MS, RI	0.1114±0.0142	0.0106±0.0009	0.000254
B4	ethyl dodecanoate	1839	1848.32	sweet waxy floral	MS, RI	0.0766±0.0052	-	0.001554
B5	methyl tetradecanoate	2020	2018.41	fatty waxy petal	MS, RI	0.1518±0.0193	0.0074±0.0008	0.000207
B6	methyl (Z)-tetradec-9-enoate	2050	2060.34	fatty waxy	MS, RI	0.173±0.006	-	9.4829E-07
B7	ethyl tetradecanoate	2057	2096.64	sweet waxy violet	MS, RI	0.1478±0.0177	-	0.004774
B8	methyl hexadecanoate	2226	2226.36	oily fatty orris	MS, RI	0.2738±0.004	0.2357±0.0191	0.068592
B9	methyl (Z)-hexadec-9-enoate	2245	2269.83	oily waxy	MS, RI	0.3113±0.0138	0.2438±0.022	0.010823
C	Alcohol							
C1	pentan-1-ol	1244	1216.53	fermented oily sweet	MS, RI	0.9649±0.0841	-	0.002525
C2	oct-1-en-3-ol	1451	1458.47	mushroom earthy green	MS, RI	0.0219±0.0004	0.029±0.0044	0.05002
C3	phenylmethanol	1898	1907.35	mild pleasant sweet	MS, RI	0.0455±0.0124	-	0.02393
C4	2-phenylethanol	1872	1926.76	floral rose phenolic	MS, RI	1.2535±0.1014	-	0.002172
C5	(1S,2R,5S,7R,8R)-2,6,6,8-tetramethyltricyclo[5.3.1.01,5]undecan-8-ol	2149	2154.94	cedarwood woody sweet	MS, RI	0.0527±0.0061	0.004±0.0002	0.00016

D	Aldehyde							
D1	nonanal	1403	1402.54	waxy aldehydic rose	MS, RI	0.0155±0.0031	-	0.000923
D2	benzaldehyde	1530	1547.31	strong bitter almond	MS, RI	-	0.0195±0.0022	0.004239
D3	2-phenylacetaldehyde	1663	1672.96	green sweet floral	MS, RI	0.0327±0.0037	-	0.004164
D4	2-benzylideneoctanal	2390	2390.52	fresh floral green	MS, RI	0.1336±0.0122	-	0.000046
E	Ketone							
E1	tridecan-2-one	1815	1819.61	fatty waxy coconut	MS, RI	0.0271±0.006	-	0.016006
E2	(E)-1-(2,6,6-trimethyl-1,3-cyclohexadien-1-yl)but-2-en-1-one	1831	1844.95	apple rose honey	MS, RI	0.3367±0.0148	-	0.00064
E3	pentadecan-2-one	2025	2032.86	fresh jasmin celery	MS, RI	0.0432±0.0037	-	0.000002
E4	6,10,14-trimethylpentadecan-2-one	2131	2135.83	oily herbal jasmin	MS, RI	0.0948±0.0064	0.0707±0.0058	0.000034

"F0" meant fermented 0h, and "F18" meant fermented 18h; odor description referred to http://www.thegoodscentscompany.com/search2.html; "-" meant not detected.

Table S3 Standard curves and detected odor threshold of 17 VOCs in F18 jujube juice.

NO.	Compound	Quantification ion	Standard Curve	R^2	F18 concentration (mg/mL)	Odor threshold (mg/mL)	OAV
A4	hexanoic acid	60, 73, 87	y = 0.3672x - 0.0094	0.9787	0.5601±0.1586	0.01409	39.7553
A5	heptanoic acid	60, 73, 87	y = 0.1439x + 0.0299	0.9659	0.4964±0.0852	0.00626	79.3034
A8	decanoic acid	60, 73, 129	y = 6.0559x - 3.1697	0.9764	5.0155±0.7592	0.22417	22.3740
A11	tetradecanoic acid	43, 60, 73	y = 5.8626x + 3.8611	0.9916	1.8321±0.2164	0.13501	13.5700
A13	dodecanoic acid	73, 129, 185	y = 3.2902x + 33.41	0.9717	13.7282±4.3498	0.12885	106.5427
B1	methyl decanoate	55, 74, 87	y = 13.385x	0.9984	0.0233±0.0009	0.01645	1.4164
B3	ethyl tetradecanoate	55, 74, 87	y = 19.426x + 1.4959	0.9898	0.213±0.0174	0.09339	2.2807
B5	methyl dodecanoate	55, 74, 87	y = 55.388x - 1.9978	0.9905	0.2172±0.045	0.08150	2.6649
B7	methyl (Z)-tetracos-15-enoate	88, 101, 157	y = 34.686x + 0.994	0.9986	0.2444±0.0106	0.46912	0.5293

B8	methyl 14-methylpentadecanoate	74, 87, 143	y = 6.5946x + 1.0329	0.9774	0.3675±0.0073	0.31764	1.1570
C1	pentan-1-ol	42, 55, 70	y = 0.057x + 0.1749	0.9824	1.5037±0.2422	0.00151	993.0731
C4	2-phenylethanol	65, 91, 122	y = 0.1591x + 0.3516	0.9665	4.2502±0.9221	0.00129	3302.3977
C5	(1R,2R,5R,7S,8R)-2,6,6,8-tetramethyltricyclo[5.3.1.01,5]undecan-8-ol	43, 95, 150	y = 28.212x + 0.5514	0.9789	0.0757±0.0037	0.00125	60.7695
D3	2-phenylacetaldehyde	65, 91, 120	y = 0.3834x + 0.0082	0.976	0.0771±0.0043	0.00009	861.6172
E2	(E)-1-(2,6,6-trimethyl-1,3-cyclohexadien-1-yl)-2-buten-1-one	69, 121, 190	y = 0.0306x + 0.0113	0.9893	0.8266±0.1185	0.00002	39828.6330
E3	pentadecan-2-one	43, 58, 71	y = 15.895x + 0.214	0.9823	0.0608±0.0052	nd	nd
E4	6,10,14-trimethylpentadecan-2-one	43, 58, 71	y = 0.8526x + 0.4345	0.9685	0.1393±0.024	nd	nd

nd, not detected, for E3 and E4 only soluble in harmful solvents.

Table S4 Details of difference compounds between F0 & F18 jujube juice.

NO.	Name	Type	Ion mode	CAS	VIP	*P*-value
1	2-Oxoglutarate	up	neg	328-50-7	1.244	0.045
2	Oxaloacetate= Oxalacetic acid	down	pos	-	1.232	0.010
3	L-Lysine;	down	pos	56-87-1	1.263	0.008
4	L-Aspartate	down	neg	56-84-8	1.263	0.005
5	Glutathione	down	neg	-	1.251	0.021
6	L-Arginine	down	pos	74-79-3	1.218	0.007
7	L-Glutamine	down	pos	56-85-9	1.257	0.005
8	L-Ornithine	down	pos	3184-13-2	1.230	0.018
9	Sucrose	down	pos	57-50-1	1.176	0.010
10	Choline	down	pos	62-49-7	1.213	0.005
11	L-Histidine	down	pos	71-00-1	1.247	0.000
12	Adenine	down	pos	73-24-5	1.264	0.001
13	L-proline	down	pos	147-85-3	1.241	0.050
14	Adenosine	down	pos	58-61-7	1.217	0.008
15	Thymidine	up	pos	50-89-5	1.216	0.016
16	Guanine	down	pos	73-40-5	1.264	0.000
17	Hypoxanthine	down	neg	68-94-0	1.255	0.005
18	Uridine	down	neg	58-96-8	1.254	0.024
19	dGMP	down	neg	902-04-5	1.230	0.016
20	Xylitol	up	neg	87-99-0	1.260	0.008
21	Cytosine	down	pos	71-30-7	1.211	0.027

22	Xanthine	down	neg	69-89-6	1.252	0.011
23	Cytidine	down	pos	65-46-3	1.079	0.105
24	Raffinose	down	pos	512-69-6	1.122	0.014
25	Deoxyadenosine	down	pos	958-09-8	1.176	0.002
26	Choline sulfate	down	pos	-	1.190	0.076
27	alpha,alpha-Trehalose	down	neg	99-20-7	1.262	0.002
28	Xylobiose	down	neg	6860-47-5	1.236	0.044
29	D-Phenylalanine	down	pos	673-06-3	1.238	0.014
30	Deoxyinosine	down	neg	69655-05-6	1.141	0.019
31	alpha-1,5-L-Arabinotriose	down	neg	-	1.209	0.062
32	alpha-1,5-L-Arabinotetraose	down	neg	-	1.240	0.000

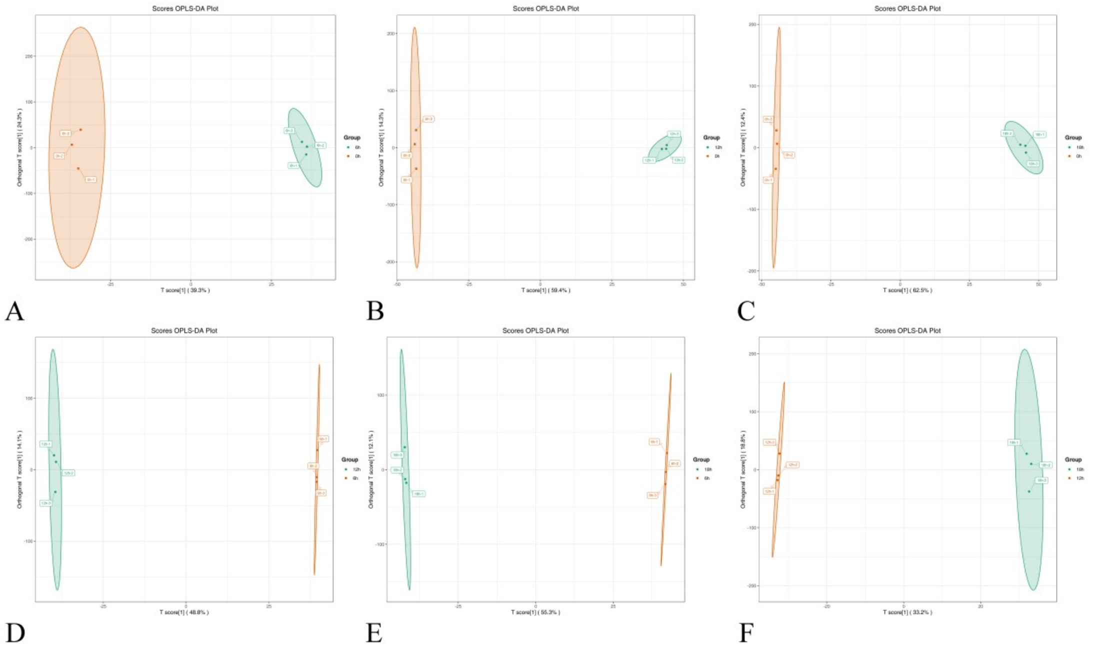

Fig. S1 (A) - (F) OPLS-DA plots, 0h vs 6h, 0h vs 12h, 0h vs 18h, 6h vs 12h, 6h vs 18h, and 12h vs 18h respectively.

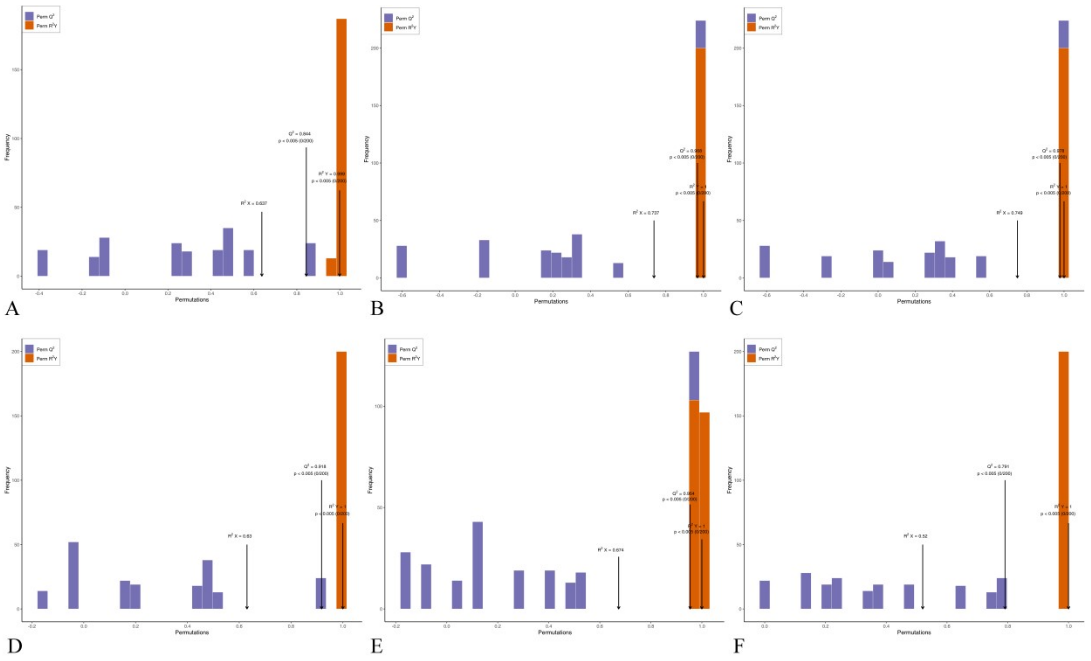

Fig. S2 (A) - (F) permutation test of OPLS-DA model, 0h vs 6h, 0h vs 12h, 0h vs 18h, 6h vs 12h, 6h vs 18h, and 12h vs 18h respectively.
